# Supplementary material for: Deep learning model DeepNeo predicts neointimal tissue characterization using optical coherence tomography
Source: Commun Med (Lond). 2025 Apr 17;5:124. doi: 10.1038/s43856-025-00835-5 (PMC12006410; doi:10.1038/s43856-025-00835-5)
Supplement: Supplementary file 2 — Supplementary Information [file 43856_2025_835_MOESM2_ESM.pdf]

Supplemental Methods

Details of segmentation network

As the segmentation networks, we evaluate a standard Unet[1], Unet++[2] and DeepLabv3[3]. We observe that Unet++ performs slightly better on average than Unet, which both perform better than DeepLabv3 as can be seen in supplementary table 1. As the best model, the Unet++ with Resnet18[4] backbone is used as the segmentation network; the network implementation in Python is taken from Iakubovskii[5]. To train the Unet++, an Adam optimizer with standard parameters is used. The initial learning rate is 0.001, to regulate learning rate, a training rate scheduler by PyTorch[6] is used which reduces the learning rate by multiplying it with a factor of 0.3 if for 5 epochs the validation loss is not decreasing. The input image resolution is 512x512 with one input channel. A batch size of 8 is used with 100 training epochs. We use the Albumentations library[7] to augment images during training. Following augmentation techniques are used: Rotate, GridDistortion, ElasticTransform, HorizontalFlip, RandomBrightnessContrast, GaussNoise, RandomGamma. As the loss function, the unweighted sum of dice loss [8], cross-entropy loss and IoU loss [9] is used.

We make use of test time augmentation to increase accuracy and make the segmentation mask invariant to rotations by 90,180 and 270 degrees as well as horizontal flipping. We do so by predicting segmentation masks for not only the initial frame but also on 90, 180 and 270 degree rotated frames on the initial as well as the horizontally flipped frame. This gives a total of eight predictions per frame, and thus eight probability distributions for each pixel of a frame, which are then fused using the mean of the distributions.

	**Lumen**	**Stent**	**Neointima**	**Average**
**Unet**	0.986 ± 0.025	0.658 ± 0.101	0.863 ± 0.135	0.837
**Unet++**	0.986 ± 0.021	0.660 ± 0.100	0.863 ± 0.138	0.838
**DeepLabv3**	0.986 ± 0.014	0.603 ± 0.110	0.868 ± 0.138	0.820

Supplemental Table 1. Comparison between segmentation performance of state-of-the art segmentation models.

Details of classification network

We found that for the classification task small models suffice. Among those, a torchvision [10] Resnet18[4] showed the best performance compared to other state-of-the art networks with the same or more parameters, implemented in the

torchvision library. Namely, we compare the performance to ViT-B[11] (smallest torchvision vision transformer) and Swin-T[12] (smallest torchvision Swin transformer) as can be seen in supplementary table 2. Note that for this comparison we measure the basic performance without test time augmentation, temperature sharpening, and without considering neighboring frames. As Resnet18 showed the best performance, it was selected as the network for DeepNeo.

	**Accuracy**	**F1-Score**
**Resnet18**	0.690	0.697
**ViT-B**	0.557	0.574
**Swin-S**	0.443	0.272

Supplemental Table 2. Comparison between classification performance of state-of-the art classification models.

To train the Resnet18, an AdamW optimizer with standard parameters is used [2]. The initial learning rate is 0.001, to regulate learning rate, a training rate scheduler by PyTorch [6] is used which reduces the learning rate by multiplying it with a factor of 0.3 if for 5 epochs the validation loss is not decreasing. The input image resolution is 224x224 with one input channel. A batch size of 32 is used with 100 training epochs. We use the Albumentations library[7] to augment images during training. Following augmentation techniques are used: GridDistortion, ElasticTransform, HorizontalFlip, RandomBrightnessContrast, GaussNoise, RandomGamma. As the loss function, cross-entropy loss is used. We also make use of test time augmentation here in a similar fashion as in the segmentation network: The same test-time augmentations are also used to generate eight class predictions per quadrant. The resulting distributions are fused using temperature sharpening and then normalizing, which yields slightly better results compared to mean aggregation and produces more meaningful confidence scores [13]. To account for spatial dependencies in a straightforward manner, in a final step we average the predictions over the surrounding quadrants:

For each quadrant k in a frame n, the final distribution $d_{k,n}^{\text{final}}$ is computed:

$$d_{k,n}^{\text{final}} = \frac{\alpha(d_{k,n-1} + d_{k,n+1}) + d_{k,n}}{2\alpha + 1} \tag{1}$$

If frame $n+1$ or $n-1$ does not exist or is outside the stented region of the pullback, the corresponding distribution gets removed in the above equation. For example, the equation would change to:

$$d_{k,n}^{\text{final}} = \frac{\alpha d_{k,n-1} + d_{k,n}}{\alpha + 1} \tag{2}$$

without frame $n+1$.

The best value for was determined via grid search on the validation set and ist set to 0.75. Accounting for surrounding frames in this manner yielded a 5.7% improvement in accuracy on the test set. The resulting confidences are well calibrated as shown in figure 4.

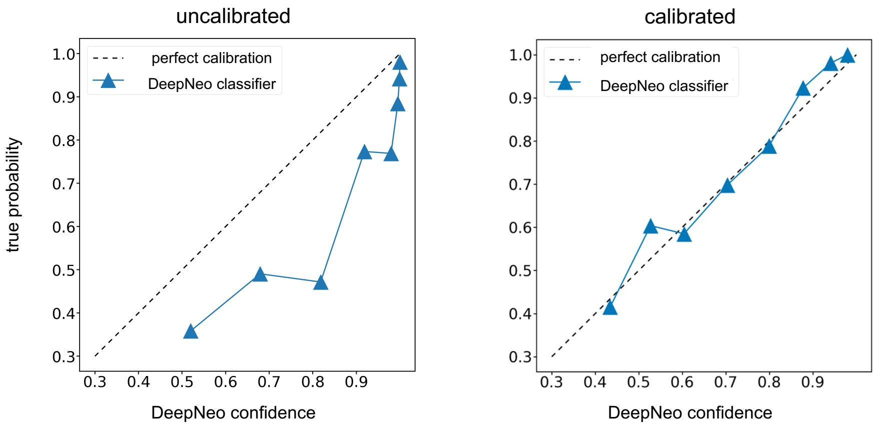

Supplemental Figure 1. Model calibration improves correlation between confidence and true probability. Uncalibrated model vs calibrated model used in DeepNeo on n=420 quadrants. Calibrated model shows a robust correlation between prediction confidence and true probability.

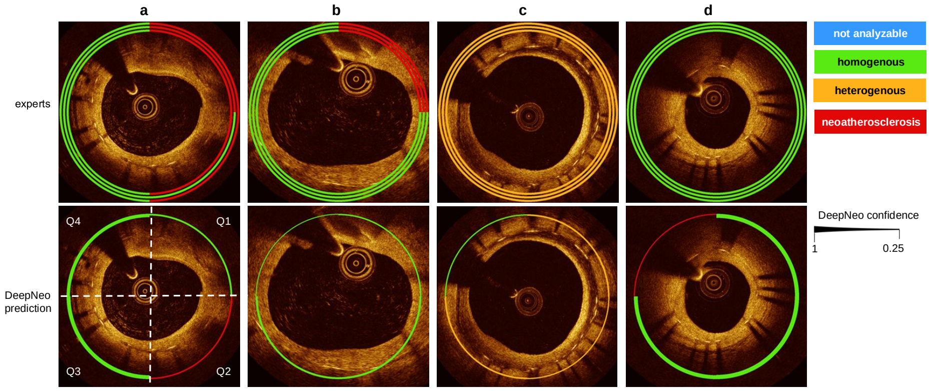

Supplemental Figure 2. Examples for prediction failure by DeepNeo. Manual annotation by three independent observers marked by three separate lines are compared to automated prediction by DeepNeo. a and b: Q1 is misclassified as homogenous. c: Q4 is misclassified as homogenous. d: Q4 is misclassified as neoatherosclerosis.

References

[1] Olaf Ronneberger, Philipp Fischer, and Thomas Brox. U-net: Convolutional networks for biomedical image segmentation. *CoRR*, abs/1505.04597, 2015.

[2] Ilya Loshchilov and Frank Hutter. Decoupled weight decay regularization, 2019.

[3] Liang-Chieh Chen, George Papandreou, Florian Schroff, and Hartwig Adam. Rethinking atrous convolution for semantic image segmentation. *CoRR*, abs/1706.05587, 2017.

[4] Kaiming He, Xiangyu Zhang, Shaoqing Ren, and Jian Sun. Deep residual learning for image recognition. *CoRR*, abs/1512.03385, 2015.

[5] Pavel Iakubovskii. Segmentation models pytorch: Segmentation models with pretrained backbones. https://github.com/qubvel/segmentation_models.pytorch. Accessed: 2024-06-10.

[6] Adam Paszke, Sam Gross, Francisco Massa, Adam Lerer, James Bradbury, Gregory Chanan, Trevor Killeen, Zeming Lin, Natalia Gimelshein, Luca Antiga, Alban Desmaison, Andreas Köpf, Edward Yang, Zach DeVito, Martin Raison, Alykhan Tejani, Sasank Chilamkurthy, Benoit Steiner, Lu Fang, Junjie Bai, and Soumith Chintala. Pytorch: An imperative style, high-performance deep learning library, 2019.

[7] Alexander V. Buslaev, Alex Parinov, Eugene Khvedchenya, Vladimir I. Iglovikov, and Alexandr A. Kalinin. Albumentations: fast and flexible image augmentations. *CoRR*, abs/1809.06839, 2018.

[8] Carole H. Sudre, Wenqi Li, Tom Vercauteren, Sebastien Ourselin, and M. Jorge Cardoso. *Generalised Dice Overlap as a Deep Learning Loss Function for Highly Unbalanced Segmentations*, page 240–248. Springer International Publishing, 2017.

[9] Hamid Rezatofighi, Nathan Tsoi, JunYoung Gwak, Amir Sadeghian, Ian Reid, and Silvio Savarese. Generalized intersection over union: A metric and a loss for bounding box regression. In *2019 IEEE/CVF Conference on Computer Vision and Pattern Recognition (CVPR)*, pages 658–666, 2019.

[10] TorchVision maintainers and contributors. Torchvision: Pytorch's computer vision library. https://github.com/pytorch/vision, 2016.

[11] Alexey Dosovitskiy, Lucas Beyer, Alexander Kolesnikov, Dirk Weissenborn, Xiaohua Zhai, Thomas Unterthiner, Mostafa Dehghani, Matthias Minderer, Georg Heigold, Sylvain Gelly, Jakob Uszkoreit, and Neil Houlsby. An image is worth 16x16 words: Transformers for image recognition at scale. *CoRR*, abs/2010.11929, 2020.

[12] Ze Liu, Yutong Lin, Yue Cao, Han Hu, Yixuan Wei, Zheng Zhang, Stephen Lin, and Baining Guo. Swin transformer: Hierarchical vision transformer using shifted windows. *CoRR*, abs/2103.14030, 2021.

[13] Chuan Guo, Geoff Pleiss, Yu Sun, and Kilian Q. Weinberger. On calibration of modern neural networks, 2017.
