# Supplementary material for: Deep learning model DeepNeo predicts neointimal tissue characterization using optical coherence tomography
Source: Commun Med (Lond). 2025 Apr 17;5:124. doi: 10.1038/s43856-025-00835-5 (PMC12006410; doi:10.1038/s43856-025-00835-5)
Supplement: Supplementary file 3 — Description of Additional Supplementary Files [file 43856_2025_835_MOESM3_ESM.pdf]

Description of Additional Supplementary Files

Supplemental data 1: Segmentation performance of DeepNeo

image: identifier of the image
class_1_dice: Dice score of class 1 (= lumen)
class_2_dice: Dice score of class 2 (=stent)
class_3_dice: Dice score of class 3 (=neointima)

Supplemental data 2: Classification performance of DeepNeo and calibration.

Image: identifier of the image
quadrant_number: Quadrant number that is analyzed
label_gt: Groundtruth neointima class as determined by the most experienced medical doctor. 0 = Not analyzable, 1= homogenous, 2= heterogenous, 3= neoatherosclerosis.
label_doc_a: Neointima class as seen by medical doctor A
label_doc_b: Neointima class as seen by medical doctor B
prediction: Prediction of DeepNeo
confidence_adjusted: Calibrated confidence of DeepNeo
confidence_unadjusted: Uncalibrated confidence of DeepNeo

Supplemental data 3: Classification performance of DeepNeo on animal cases

animal_nr: identifier of the animal
section: section identifier of the probe
quadrant: Quadrant number that is analyzed
histo_class: Neointima class as determined by a specialist according to the histopathological image. 0 = Not analyzable, 1= homogenous, 2= heterogenous, 3= neoatherosclerosis
deepneo_prediction: Prediction by DeepNeo
